# Physical activity as a risk or protective factor for falls and fall-related fractures in non-frail and frail older adults: a longitudinal study

**DOI:** 10.1186/s12877-022-03383-y

**Published:** 2022-08-22

**Authors:** Maaike van Gameren, Emiel O. Hoogendijk, Natasja M. van Schoor, Daniël Bossen, Bart Visser, Judith E. Bosmans, Mirjam Pijnappels

**Affiliations:** 1 Department of Human Movement Sciences, Vrije Universiteit Amsterdam, Amsterdam, the Netherlands; 2 Amsterdam Movement Sciences, Amsterdam, the Netherlands; 3 Department of Epidemiology and Data Science, Amsterdam University Medical Center, Vrije Universiteit Amsterdam, Amsterdam, the Netherlands; 4 Amsterdam Public Health, Amsterdam, the Netherlands; 5 Faculty of Health, Centre of Expertise Urban Vitality, Amsterdam University of Applied Sciences, Amsterdam, the Netherlands; 6 Department of Health Sciences, Faculty of Science, Vrije Universiteit Amsterdam, Amsterdam, the Netherlands

**Keywords:** Accidental falls, Fall-related injuries, Frail older adults, Aging, Accelerometry, Fall risk

## Abstract

**Background:**

Physical activity may be both a risk and protective factor for falls and fall-related fractures. Despite its positive effects on muscle and bone health, physical activity also increases exposure to situations where falls and fractures occur. This paradox could possibly be explained by frailty status. Therefore, the aim of this study was to investigate the associations between physical activity and both falls and fractures, and to determine whether frailty modifies the association of physical activity with falls, and fractures.

**Methods:**

Data of 311 community-dwelling participants aged 75 years or older from the Longitudinal Aging Study Amsterdam, who participated in a three-year longitudinal study with five nine-monthly measurements between 2015/2016 and 2018/2019. Their mean age was 81.1 (SD 4.8) years and frailty was present in 30.9% of the participants. Physical activity in minutes per day was objectively assessed with an inertial sensor (Actigraph) for seven consecutive days. Falls and fractures were assessed every nine months using self-report during an interview over a follow-up period of three years. Frailty was determined at baseline using the frailty index. Associations were estimated using longitudinal logistic regression analyses based on generalized estimating equations.

**Results:**

No association between physical activity and falls was found (OR = 1.00, 95% CI: 0.99–1.00). Fall risk was higher in frail compared to non-frail adults (OR = 2.21, 95% CI: 1.33–3.68), but no effect modification was seen of frailty on the association between physical activity and falls. Also no relation between physical activity and fractures was found (OR = 1.00, 95% CI: 0.99–1.01). Fracture risk was higher in frail compared to non-frail adults (OR = 2.81, 95% CI: 1.02–7.75), but also no effect modification of frailty was present in the association between physical activity and fractures.

**Conclusions:**

No association between physical activity and neither falls nor fractures was found, and frailty appeared not to be an effect modifier. However, frailty was a risk factor for falls and fractures in this population of older adults. Our findings suggest that physical activity can be safely recommended in non-frail and frail populations for general health benefits, without increasing the risk of falls.

**Supplementary Information:**

The online version contains supplementary material available at 10.1186/s12877-022-03383-y.

## Background

Falls are one of the major causes of mortality and morbidity in older adults of 65 years or older [1]. More than one-third of the population aged 65 years or older falls at least once each year [1, 2]. Major injuries, such as head trauma and fractures occur in 10–15% of falls [1, 2]. Physical inactivity is suggested to be a risk factor for falls and fall-related fractures in older persons due to its negative effects on gait, balance control, mobility, and muscle strength [3–5]. Previous research has indeed shown that high levels of physical activity could be associated with a lower risk of falls and fractures in older adults, because physical activity helps to maintain balance control and muscle and bone strength [6–9]. However, high levels of physical activity may also increase the risk of both falls and fall-related fractures [10, 11]. A longer duration [12–14] or intensity [15, 16] of physical activity increases exposure to situations where falls, and thus fractures, could occur. Thus, there is a paradox regarding the question whether physical activity is a risk factor or a protective factor for falls and fall-related fractures [10, 17]. 

This paradox could possibly be explained by frailty status [18, 19]. Frailty is a state of increased vulnerability to adverse outcomes resulting from low physiological reserves, low resistance to stressors and multisystem impairment [20–22]. Whether and how frailty affects the association between physical activity and both falls and fractures is not clear and pathways may be complex considering the multidimensionality of frailty and the numerous risk factors for falls and fractures [18, 19]. The frailty index is a measure of frailty and involves the accumulation of diseases, activities of daily living, and cognitive and psychological function disabilities, in which a greater number of health deficits indicates a higher frailty status [23]. One possible explanation for an increased fall risk in frail older adults compared to non-frail older adults is that sarcopenia (i.e., lower muscle mass and strength) is a major component of the frailty status [24, 25]. Therefore, physical activity in frail older adults is possibly associated with more falls compared to non-frail older adults, due to reduced muscle strength [18, 19, 26].

Current clinical guidelines and health care policies for older persons recommend physical activity because of its beneficial effects on many health outcomes [27, 28], but these guidelines do not take frailty status into account. An adverse effect of these recommendations may be increased fall and fracture incidences among frail older adults. However, it is also possible that high physical activity is associated with more falls, but with less fractures among frail older adults, because of an increased bone strength [29]. In that case, the positive health outcomes of physical activity may outweigh the consequences of a fall. Therefore, a better understanding of the complex relationship between physical activity and falls, and physical activity and fall-related fractures is warranted.

The aims of this study were to investigate the associations between physical activity and falls, and physical activity and fall-related fractures, and to examine whether frailty is an effect modifier of both associations in a population of older adults. Since physical activity helps to maintain balance control and muscle and bone strength, but leads to increased exposure to situations where falls occur, physical activity was expected to increase fall incidence among both non-frail older adults and frail older adults [6–8]. The fracture risk was expected to decrease among non-frail, but to increase in frail older adults with increased duration of physical activity [8].

## Methods

### Study design and participants

This study was performed using data from the Longitudinal Aging Study Amsterdam (LASA), an ongoing cohort study on physical, cognitive, emotional and social functioning in older adults to determine predictors and consequences of ageing [30]. The data collection procedures have been described in detail elsewhere [31, 32].

For the current study, data were used from the LASA 75 PLUS study, i.e. five measurement waves over a period of three years (time point 1, T1: baseline, 2015/2016, time point 2, T2: nine months after baseline, time point 3, T3: 18 months after baseline, time point 4, T4: 27 months after baseline and time point 5, T5: 3 years after baseline, 2018/2019) [33]. A total of 601 participants agreed to participate. Because of missing accelerometry data and data of the LASA Physical Activity Questionnaire, final analysis in this longitudinal study included a representative study population of 311 participants aged 75 years or older with complete data. The Medical Ethics Committee of the VU University Medical Centre approved the study. All participants in this study signed informed consent. This study was conducted according to the principles of the Declaration of Helsinki (7th revision, October 2013) and is performed in accordance with the Medical Research Involving Human Subjects Act (WMO) and other guidelines, regulations and acts such as Good Clinical Practice and the statement conducting research involving humans.

### Baseline characteristics

At T1, information was collected about the age, sex, and BMI of the participants. Moreover, dizziness complaints were determined by the question whether participants are dizzy regularly (yes/no). Furthermore, the six-meter walking time was assessed by asking subjects to walk three meters, to turn around and walk back three meters as quickly as possible. Based on this assessment, an average walking speed was calculated. Last, grip strength was determined by using a grip strength dynamometer (JAMAR 5030J1 Hydraulic Hand Dynamometer). Participants were asked to perform the grip strength exercise twice with each hand. Grip strength was determined as the average of the highest score of the left and the right hand. The position of the participants was seated with the back straight and elbow bended in a 90° angle. The dynamometer was adjusted for hand size.

### Falls and fractures

A fall was defined as ‘an unintentional change in position resulting in coming to rest at a lower level or on the ground’ [34]. From T1 up to and including T5, participants kept a fall and fracture diary and were interviewed over telephone every nine months about falls and fractures. If a fall was reported over the past nine months, participants were asked whether a fracture resulted from their fall.

### Physical activity

To measure physical activity objectively, participants were sent an Actigraph tri-axial inertial sensor (Model GT3X; Actigraph, Pensacola, FL, USA) by mail together with the instructions for wearing the inertial sensor at T1. The inertial sensor was attached to a three centimetre wide, tight elastic belt and was worn superior to the left iliac crest. Two days after sending the inertial sensor, a phone call to the participant was made to ensure that the package was received and the inertial sensor was properly worn. Participants were instructed to wear the inertial sensor for seven consecutive days during waking hours and to remove the inertial sensor only during bathing, showering and swimming. Participants kept a diary to log the time the inertial sensor was put on after waking and removed before sleeping. When the inertial sensor was not worn for some period during the day, participants recorded the start and end time of the period not wearing the inertial sensor. Physical activity was defined as the time spent on at least light-high intensity in minutes per day, defined as the inertial sensor registering at least 760 counts per minute (a count is a relative measure of change in momentum as measured in 3D by the inertial sensor) [35–37].

### Frailty

At T1, frailty was determined using the frailty index, which is a valid and reliable instrument to determine frailty in older adults [23, 38, 39]. The frailty index includes 32 health deficits from physical, mental and cognitive domains. These health deficits include self-reported chronic conditions (11 items), functional limitations (six items), self-rated health (two items), mental health (six items from the Centre for Epidemiologic Studies Depression Scale), physical activity (one item), cognitive health (five items, based on self-reported memory complaints and domains of the Mini-Mental State Examination), and physical performance measured by gait speed (one item) [23, 40, 41]. All deficits were scored as 0 or 1, where 0 indicates the absence of the deficit and 1 the presence of the deficit. The frailty score was not calculated if participants had more than 20% missing items. This criterion is commonly used and allows for maximum use of available data without excessive reliance on imputation procedures [42]. A frailty score was calculated for each participant by dividing the sum of the health deficit scores by the total number of health deficits assessed. This resulted in a score between 0 (no deficits present) and 1 (all deficits present). Participants were considered to be non-frail if they had a frailty index score < 0.25 and were classified as frail when having a frailty index score ≥ 0.25 [43].

### Statistical analysis

Data were analysed using IBM SPSS Statistics version 27 (IBM Corp. Armonk, NY) and RStudio version 1.3.1073 (RStudio Team. Boston, MA). *P*-values were based on two-sided tests and were a priori considered statistically significant at *p* < 0.05 a priori and not less than or equal to 0.05.

#### Descriptive statistics

To describe the study population at baseline, descriptive statistics (mean, median, SD, IQR) were calculated while stratifying for frailty status. Differences in baseline characteristics between non-frail and frail participants were analysed using Chi-squared tests and Mann–Whitney U tests since all continuous variables were skewed. Differences in the daily duration of physical activity between fallers and non-fallers, participants who experienced a fracture and participants who experienced no fracture, and frail and non-frail participants were estimated by Mann–Whitney U tests since physical activity was non-normally distributed.

#### Generalized estimating equations

To examine the associations between physical activity on the one hand and falls and fall-related fractures on the other hand, we used generalized estimating equations (GEE’s) with longitudinal fall and fracture data over the period of 3 years. In these analyses, all data available were included in the models to prevent a healthy survivor effect. The GEE models take into account the dependency between repeated measures within a subject [44]. The GEE analyses were estimated using an exchangeable correlation matrix. For both falls and fall-related fractures as outcome measures, we analysed four models. In the first model, the association between physical activity and falls or fractures was examined. In the second model, the association between frailty and falls or fractures was determined. In the third model, physical activity, frailty and an interaction term of physical activity and frailty were included to determine whether frailty status was an effect modifier. This was done by checking whether the interaction term was statistically significant. In the fourth model, age and sex were added as covariates to model 3. We tested for a non-linear association between physical activity and falls or fractures by adding a quadratic term for physical activity, but this was not statistically significant and therefore not included in the final models. Odds ratios were estimated as well as 95% confidence intervals and *p*-values.

#### Sensitivity analyses

Two sensitivity analyses were performed to test for the robustness of our findings in a larger population with less specific data. In a first sensitivity analysis, the LASA Physical Activity Questionnaire (LAPAQ) was used to assess physical activity [45]. This allowed for a sensitivity analysis on 505 subjects participating in the LASA 75 PLUS study of whom LAPAQ data was available, and thus on a larger power compared to the primary analysis using inertial sensor data. The LAPAQ subjectively assesses the frequency and duration of activities in the past 14 days. The activities included were walking, cycling, heavy household work and a first and second sport when applicable. The frequency of the activity was multiplied by the duration of the activity in minutes per day and then divided by 14 days (frequency*duration/14).

In a second sensitivity analysis, binary logistic regression analyses were performed in an even larger study population of 1,002 participants of 65 years or older to further increase the power of the analyses and test for robustness of our primary findings. These regression analyses included the same four models as in the primary analyses. For this cross-sectional study, data were used from T1 (2015/2016) and T5 (2018/2019). This study population consisted of participants aged65 years and older and was larger than the primary study population, because falls and fractures were retrospectively asked over the past 3 years instead of determined every nine months. In this population, physical activity was determined at baseline by an Actigraph tri-axial inertial sensor (Model GT3X; Actigraph, Pensacola, FL, USA).

## Results

**Fig. 1:**
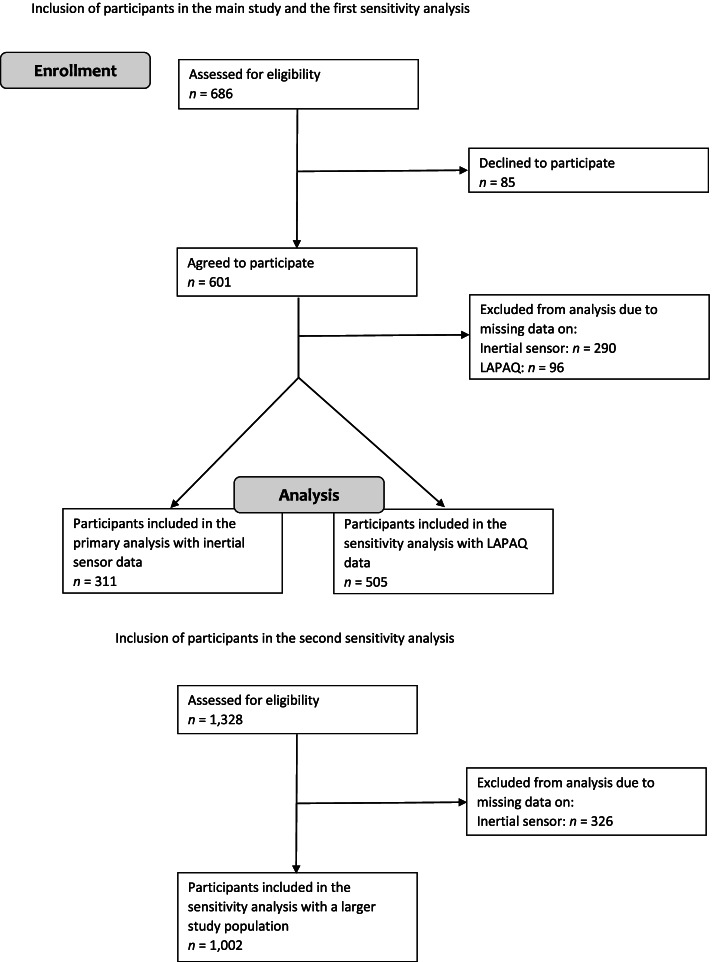
The inclusion process of the study

Between 2016 and 2019, 686 potential participants aged 75 years or older were invited to participate in this study. A total of 601 participants (87.6%) agreed to participate. Final analysis was performed on 311 participants who wore the inertial sensor. A first sensitivity analysis was done on 505 participants with available LAPAQ data and a second sensitivity analysis was done on 1,002 participants with inertial sensor data but longer follow-up on falls and fractures. The inclusion process of the study is shown in Fig. 1.

Table 1 shows the characteristics of the study population at baseline, stratified for non-frail (*n* = 215) and frail (*n* = 96) older adults. Among these 311 subjects, the mean age in the total sample was 81.1 (SD 4.8) years and the majority were women (*n* = 173, 55.6%). The mean time spent on physical activity in the total sample was 10.8 (SD 14.7) minutes per day as assessed by the Actigraph and 54.5 (SD 52.7) minutes per day as assessed by the LAPAQ. The mean Body Mass Index was 27.2 (SD 4.1). A total of 51 participants (12.5%) reported feeling dizzy regularly. Mean six-meter walking speed with a turn at three meter was lower in the non-frail group compared to the frail group, and grip strength was higher in the non-frail group compared to the frail group.

**Table 1:** Baseline characteristics of the study population

	Non-frail *n* = 215	Frail *n* = 96	*p* value
Age (years)^a^	79.0 (76.9–82.7)	81.8 (79.1–86.9)	< 0.001
Sex (female)^b^	113 (52.6%)	60 (62.5%)	0.10
BMI^a^	25.7 (23.8–29.1)	28.1 (25.8–30.8)	< 0.001
Dizziness (yes)^b^	33 (15.3%)	18 (18.8%)	0.30
Frailty index score^a^	0.16 (0.11–0.20)	0.32 (0.27–0.38)	< 0.001
Mean six-meter walking speed (m/sec)^a, c^	7.0 (6.0–8.0)	11.0 (8.0–13.5)	< 0.001
Grip strength (kg)^a^	24 (18.1–32.4)	18.5 (13.5–25.5)	< 0.001
Physical activity (minutes per day)^a, d^	55.1 (31.1–87.3)	25.9 (9.5–51.0)	< 0.001
LAPAQ^a, d^	58.6 (30.0–88.6)	27.1 (11.8–45.0)	< 0.001

*BMI* Body Mass Index, *LAPAQ* LASA Physical Activity Questionnaire, *IQR* Interquartile range

*P* values are calculated using Chi-squared tests and Mann–Whitney U tests

^a^ Presented as median (IQR)

^b^ Presented as *n* (percentage)

^c^ Six meter walking time was tested by asking subjects to walk 3 m, to turn around and walk back 3 m as quickly as possible in m/sec

^d^ Duration of physical activities at least at light-high intensity

### Physical activity and falls

**Fig. 2:**
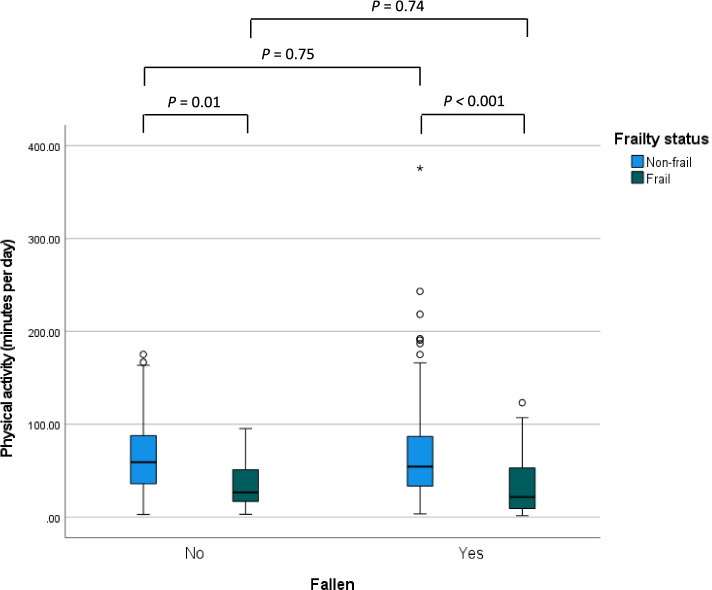
Daily amount of physical activity at least at light-high intensity for participants who did and did not experience a fall, stratified for non-frail (*n* = 215) and frail (*n* = 96) older adults

Figure 2 shows the amount of physical activity for participants who did and did not experience a fall during follow-up, stratified for non-frail and frail older adults. Among non-fallers, the mean daily amount of physical activity among non-frail adults was 66.7 min (SD 41.5) and was significantly higher than among frail adults with 35.2 (SD 26.1) daily minutes of physical activity per day (95% CI of the difference: 7.8 to 55.3 min per day). Among fallers, the mean daily amount of physical activity among non-frail adults was 69.1 min (SD 54.3) and was significantly higher than frail adults with 32.3 (SD 29.0) daily minutes of physical activity per day (95% CI of the difference: 25.0 to 48.6 min per day). No difference in physical activity was found between fallers and non-fallers with the same frailty status.

Model 1 of the unadjusted GEE analyses in Table 2 shows no association between physical activity and falls. Model 2 shows an increased fall risk in frail older adults compared to non-frail older adults, with an adjusted odds ratio of 1.71 (95% CI: 1.33 – 2.20). The association between frailty and falls is also significant without the interaction term in the model (data not shown). In model 3, no interaction between physical activity and frailty was found, thus frailty appeared not to be an effect modifier in the association between physical activity and falls. Adjustment for age and sex in model 4 had negligible impact on the association between physical activity, frailty and falls (Model 3 versus Model 4, Table 2).

**Table 2:** Generalized estimating equation models for falls

	Model 1: physical activity			Model 2: frailty			Model 3: physical activity, frailty and interaction term			Model 4: physical activity, frailty, age and sex		
	**OR**	**95% CI**	***p* value**	**OR**	**95% CI**	***p* value**	**OR**	**95% CI**	***p* value**	**OR**	**95% CI**	***p* value**
Physical activity (minutes/day)	1.00	0.99–1.00	0.26				1.00	1.00–1.00	0.65	1.00	1.00–1.01	0.39
Frailty												
Non-frail Frail				Ref 1.71	1.33–2.20	< 0.001	Ref 2.21	1.33–3.68	0.002	Ref 1.69	1.19–2.40	0.004
Physical activity * frailty							1.00	1.00–1.00	0.27			
Age										1.04	1.00–1.07	0.06
Sex												
Men Women										Ref 1.30	0.94–1.80	0.12

*OR* Odds ratio, *CI* Confidence Interval, *Ref* Reference group. Analysis included 311 respondents and 1144 observations

### Physical activity and fall-related fractures

**Fig. 3:**
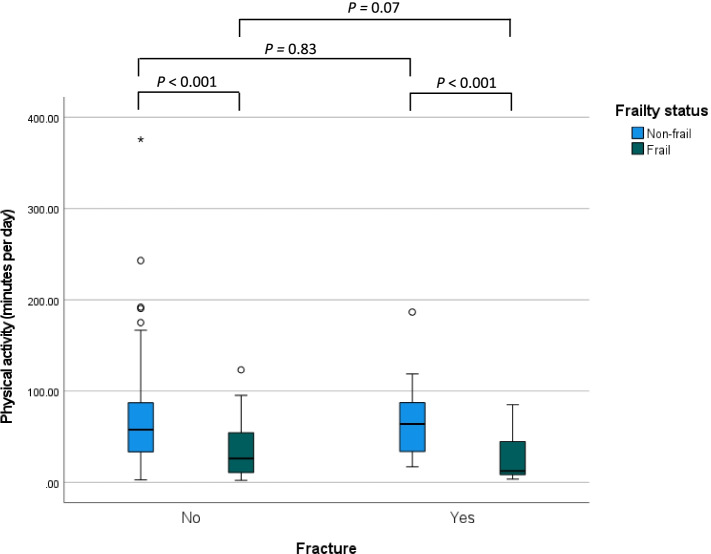
Amount of physical activity at least at light-high intensity per day for participants who did and did not experience a fracture during follow-up, stratified for non-frail (*n* = 215) and frail (*n* = 96) older adults

Figure 3 shows the amount of physical activity for participants who did and did not experience a fracture, stratified for non-frail and frail older adults. Among participants who did not experience a fracture, the mean amount of physical activity for non-frail adults was 68.4 (SD 50.6) minutes and was significantly higher compared to frail older adults with 34.8 (SD 28.0) daily minutes of physical activity per day (95% CI of the difference: 28.7 to 50.4 min per day). Among participants who experienced a fracture, the mean daily amount of physical activity among non-frail adults was 67.1 (SD 39.3) minutes and was significantly higher compared to frail older adults with 27.6 (SD 25.1) daily minutes of physical activity per day (95% CI of the difference: -0.66 to 15.11 min per day). No difference in amount of physical activity was found between participants who experienced a fracture and participants who did not experienced a fracture with the same frailty status.

Model 1 of the unadjusted GEE analyses in Table 3 shows no association between physical activity and fall-related fractures. Model 2 shows an increased risk of fall-related fractures in frail compared to non-frail older adults with an adjusted odds ratio of 2.10 (95% CI: 1.18—3.75). The association between frailty and fractures is not significant without the interaction term in the model (data not shown). In model 3, no interaction between physical activity and frailty has been found, thus frailty appeared not to be an effect modifier in the association between physical activity and fractures. Adjustment for age and sex in model 4 had negligible impact on the association between fractures, physical activity, frailty and the interaction term (Model 3 versus Model 4, Table 3).

**Table 3:** Generalized estimating equation models on fall-related fractures

	Model 1: physical activity			Model 2: frailty			Model 3: physical activity, frailty and interaction term			Model 4: physical activity, frailty, age and sex		
	**OR**	**95% CI**	***p* value**	**OR**	**95% CI**	***p* value**	**OR**	**95% CI**	***p* value**	**OR**	**95% CI**	***p* value**
Physical activity (minutes/day)	1.00	0.99–1.01	0.72				1.00	0.99–1.01	0.56	1.00	0.99–1.01	0.83
Frailty												
Non-frail Frail				Ref 2.10	1.18–3.75	0.01	Ref 2.81	1.02–7.75	0.05	Ref 1.90	0.92–3.90	0.08
Physical activity * frailty							0.99	0.97–1.01	0.33			
Age										1.00	0.94–1.08	0.92
Sex												
Men Women										Ref 1.05	0.51–2.19	0.89

*OR* Odds ratio, *CI* Confidence Interval, *Ref* Reference group. Analysis included 311 respondents and 1144 observations

### Sensitivity analyses

#### LASA physical activity questionnaire

As a sensitivity analysis, the LASA Physical Activity Questionnaire (LAPAQ) was used to define physical activity. This sensitivity analysis was performed in 505 participants, and thus had a larger power compared to the primary analysis. Age, physical activity and frailty were comparable to the study population of the primary analysis. Results were similar to the primary analysis (Table 1 and 2 in the Supplementary Material). 

#### Larger study population

In a second sensitivity analysis, the primary analysis was repeated in a larger study population of 1,002 participants of 65 years or older to further increase the power of the analyses. This sensitivity analysis was conducted in a larger study population to evaluate the robustness of the study results. The mean amount of physical activity was 88.4 min per day. Frailty index scores were similar compared to the study population of the primary analysis. Results were similar to the primary analysis (Table 3 and 4 in the Supplementary Material).

## Discussion

This is the first study that examined the moderating role of frailty in the association between physical activity and falls, and physical activity and fractures among community-dwelling older adults. We hypothesized to find a paradox in that PA would have a different relation with falls or fractures in frail compared to non-frail older adults, but in fact, we did not find a relation between PA and either falls nor fractures. Moreover, frailty did not modify both associations, but was associated with an increased fall and fracture risk. Sensitivity analyses in a larger study population found comparable results and thus substantiated the results of the primary analysis. Therefore, we cannot confirm our hypothesis that physical activity is a risk factor for falls in all older adults and fractures in frail older adults, and is protective for fractures in non-frail older adults.

In contrast to our results, an association between physical activity and fall risk was previously found and showed that physical activity can both increase and decrease fall risk [9, 11, 14, 46]. An explanation for not finding an association between physical activity and fall risk is that physical activity levels of our participants showed little variance. Therefore, participants with the highest and lowest physical activity levels were possibly underrepresented, causing an underestimation of the association between physical activity and falls. Another possible explanation is that some studies reported that men have an increased fall risk and women a decreased fall risk when being more physically active [4, 47]. However, when we added sex as a covariate in the analyses, this appeared not to affect the association between physical activity and falls.

We also did not find an association between physical activity and fall-related fractures in our study, in contrary to previous research [48–50]. However, other studies also did not find a significant correlation between physical activity and fall-related fractures [11, 51, 52]. These results may be explained by only long-term physical activity for more than one year leading to a reduction in fracture risk [11]. Moreover, in this study we did not take the intensity of the physical activity into account. Possibly, being physical active on a high intensity results in more falls and thus fractures compared to physical activity on a low intensity. On the other hand, when exercising on a higher intensity, the bone density could increase and thus reduces the fall risk [53]. Therefore, it would be interesting to investigate the intensity of physical activity on falls and fractures in further research.

To our best knowledge, no previous research has been conducted on the modifying effect of frailty on the relationship between physical activity and both falls and fall-related fractures. Moreover, frailty did not modify the association of physical activity with fall risk and fall-related fractures. However, similar as found in this study, frail older adults have been shown less physically active and have a higher risk of falls and fall-related fractures compared to non-frail adults [54–57]. Moreover, high physical activity has been shown related to more falls, but only among women impaired in their instrumental activities in daily living tasks [58]. This suggests that frailty is possibly a more important factor than physical activity when predicting falls and fall-related fractures. Further research should investigate this.

A strength of this study is the participation of a large sample of nationally representative data from a large study among community-dwelling older adults in the Netherlands of 75 years or older. Another strength of this study is that physical activity was assessed using inertial sensors, resulting in objective measures of the duration of physical activity, in contrast to questionnaires that were frequently used in previous research and often result in an overestimation. Furthermore, falls and fractures were prospectively determined by keeping a fall and fracture diary, and were assessed every 9 months by telephone calls with the researchers. Last, two sensitivity analyses were conducted; both showed similar results as the primary analyses, which substantiated the results found in this study.

The current study is limited by the absence of physical activity and frailty data at the moment of follow-up. It may be that the level of physical activity and frailty status changed during the three-year follow-up period, affecting the fall risk. Second, the amount of falls and fractures in this study population with 311 participants was limited. Therefore, it is more difficult to adequately test for interaction effects, because of a limited power. Further research is recommended to include a larger study population with more events. However, when conducting sensitivity analyses in a larger study population, results were the same compared to the primary analysis. Last, as in all longitudinal studies, there is the risk of subject attrition, which can lead to a motivated and healthy study sample, and an overestimation of physical activity and an underestimation of frailty, falls and fractures. To minimize a healthy survivor effect, we took all available data into account in the GEE analyses and not only the cases having complete data.

The results of our study have implications for clinical practice and public health. In this study, we found that more physical activity does not decrease, but does also not increase fall and fracture risk. Since physical activity has major health benefits, such as more muscle and bone strength, but also a reduced risk of, for example, cardiovascular disease and diabetes, the advice remains to encourage older adults to be physically active [59]. Because frailty appeared related to falls or fractures in our population, frail older adults should be monitored. Because of the ageing population, the number of older adults is increasing, of which a growing proportion will be frail. As frailty does not modify the interaction between physical activity and both falls and fractures, also in this group of frail older adults the advice remains to stay physically active. Thus, frailty is important for fall and fractures risk, but not specifically in the context of physical activity. Further research should investigate the extent and intensity to which physical activity is safe for frail older adults taken into account their higher risk of falls. Besides, a broader approach is needed to prevent falls and fractures than only looking at physical activity, when acting on all aspects of frailty.

## Conclusion

Longer durations of physical activity did not decrease or increase the risk of falls or fractures in our sample of community-dwelling adults of 75 years or older. However, frail older adults in our study did have an increased fall and fracture risk compared to the non-frail participants.

## Supplementary Information

**Additional file 1: Supplementary Table 1*.*** Sensitivity analysis on fall risk using the LAPAQ as definition for physical activity*. ***Supplementary Table 2*.*** Sensitivity analysis on fall-related fractures using the LAPAQ as definition for physical activity*. ***Supplementary Table 3*.*** Sensitivity analysis on fall risk using inertial se*nsor data for physical activity. ***Supplementary Table 4*.*** Sensitivity analysis on the risk of fall-related fractures using inertial sensor data for physical activity.

## Data Availability

Only the investigators had access to the pseudonymised final full trial dataset. The data are available for analysis by external researchers and can be requested (see www.lasa-vu.nl for data request procedures).

## Abbreviations

BMI: Body mass index
GEE: Generalized estimating equations
LASA: Longitudinal Aging Study Amsterdam
LAPAQ: LASA Physical Activity Questionnaire
OR: Odds ratio
