# Supplementary material for: Physical activity as a risk or protective factor for falls and fall-related fractures in non-frail and frail older adults: a longitudinal study
Source: BMC Geriatr. 2022 Aug 22;22:695. doi: 10.1186/s12877-022-03383-y (PMC9396867; doi:10.1186/s12877-022-03383-y)
Supplement: Supplementary file 1 — Additional file 1: Supplementary Table 1. Sensitivity analysis on fall risk using the LAPAQ as definition for physical activity. Supplementary Table 2. Sensitivity analysis on fall-related fractures using the LAPAQ as definition for physical activity. Supplementary Table 3. Sensitivity analysis on fall risk using inertial sensor data for physical activity. Supplementary Table 4. Sensitivity analysis on the risk of fall-related fractures using inertial sensor data for physical activity. [file 12877_2022_3383_MOESM1_ESM.docx]

**Additional file 1**

**Results of the sensitivity analyses**

**Sensitivity analysis with the LAPAQ used to define physical activity with falls as outcome measure**

**Supplementary Table 1 Sensitivity analysis on fall risk using the LAPAQ as definition for physical activity**

|  | Model 1: physical activity | | | Model 2: frailty | | | Model 3: physical activity, frailty and interaction term | | | Model 4: physical activity, frailty, age and sex | | |
| --- | --- | --- | --- | --- | --- | --- | --- | --- | --- | --- | --- | --- |
|  | **OR** | **95% CI** | ***p* value** | **OR** | **95% CI** | ***p* value** | **OR** | **95% CI** | ***p* value** | **OR** | **95% CI** | ***p* value** |
| Physical activity (minutes/day) | 1.00 | 1.00-1.00 | 0.89 |  |  |  | 1.00 | 1.00-1.00 | 0.46 | 1.00 | 1.00-1.01 |  |
| Frailty  Non-frail  Frail |  |  |  | Ref 1.71 | 1.33-2.20 | <0.001 | Ref 1.66 | 1.16-2.39 | 0.01 | Ref 1.64 | 1.25-2.14 | <0.001 |
| Physical activity * frailty |  |  |  |  |  |  | 1.00 | 1.00-1.00 | 0.59 |  |  |  |
| Age |  |  |  |  |  |  |  |  |  | 1.04 | 1.01-1.06 | 0.005 |
| Sex  Men  Women |  |  |  |  |  |  |  |  |  | Ref 1.06 | 0.81-1.39 | 0.65 |

**OR Odds ratio, CI Confidence Interval, Ref Reference group. Analysis included 504 respondents and 1752 observations.**

**Sensitivity analysis with the LAPAQ used to define physical activity with fall-related fractures as outcome measure**

**Supplementary Table 2 Sensitivity analysis on fall-related fractures using the LAPAQ as definition for physical activity**

|  | Model 1: physical activity | | | Model 2: frailty | | | Model 3: physical activity, frailty and interaction term | | | Model 4: physical activity, frailty, age and sex | | |
| --- | --- | --- | --- | --- | --- | --- | --- | --- | --- | --- | --- | --- |
|  | **OR** | **95% CI** | ***p* value** | **OR** | **95% CI** | ***p* value** | **OR** | **95% CI** | ***p* value** | **OR** | **95% CI** | ***p* value** |
| Physical activity (minutes/day) | 1.00 | 0.99-1.00 | 0.23 |  |  |  | 1.00 | 0.99-1.00 | 0.21 | 1.00 | 0.99-1.00 | 0.54 |
| Frailty  Non-frail  Frail |  |  |  | Ref 2.10 | 1.18-3.75 | 0.01 | Ref 1.49 | 0.66-3.33 | 0.33 | Ref 2.13 | 1.11-4.07 | 0.02 |
| Physical activity * frailty |  |  |  |  |  |  | 1.01 | 1.00-1.02 | 0.22 |  |  |  |
| Age |  |  |  |  |  |  |  |  |  | 0.99 | 0.94-1.05 | 0.83 |
| Sex  Men  Women |  |  |  |  |  |  |  |  |  | Ref 0.82 | 0.45-1.49 | 0.51 |

**OR Odds ratio, CI Confidence Interval, Ref Reference group. Analysis included 504 respondents and 1752 observations.**

**Sensitivity analysis with inertial sensor data used to define physical activity with falls as outcome measure**

**Supplementary Table 3 Sensitivity analysis on fall risk using inertial sensor data for physical activity**

|  | Model 1: physical activity | | | Model 2: frailty | | | Model 3: physical activity, frailty and interaction term | | | Model 4: physical activity, frailty, age and sex | | |
| --- | --- | --- | --- | --- | --- | --- | --- | --- | --- | --- | --- | --- |
|  | **OR** | **95% CI** | ***p* value** | **OR** | **95% CI** | ***p* value** | **OR** | **95% CI** | ***p* value** | **OR** | **95% CI** | ***p* value** |
| Physical activity (minutes/day) | 1.00 | 0.99-1.00 | 0.07 |  |  |  | 1.00 | 1.00-1.00 | 0.42 | 1.00 | 1.00-1.00 | 0.78 |
| Frailty  Non-frail  Frail |  |  |  | Ref 1.86 | 1.37-2.54 | <0.001 | Ref 1.89 | 1.03-3.50 | 0.04 | Ref 1.66 | 1.12-2.47 | 0.01 |
| Physical activity * frailty |  |  |  |  |  |  | 1.00 | 0.99-1.01 | 0.93 |  |  |  |
| Age |  |  |  |  |  |  |  |  |  | 1.04 | 1.01-1.07 | 0.006 |
| Sex  Men  Women |  |  |  |  |  |  |  |  |  | Ref 1.69 | 1.24-2.30 | 0.001 |

**OR Odds ratio, CI Confidence Interval, Ref Reference group.**

**Sensitivity analysis with inertial sensor data used to define physical activity with fall-related fractures as outcome measure**

**Supplementary Table 4 Sensitivity analysis on the risk of fall-related fractures using inertial sensor data for physical activity**

|  | Model 1: physical activity | | | Model 2: frailty | | | Model 3: physical activity, frailty and interaction term | | | Model 4: physical activity, frailty, age and sex | | |
| --- | --- | --- | --- | --- | --- | --- | --- | --- | --- | --- | --- | --- |
|  | **OR** | **95% CI** | ***p* value** | **OR** | **95% CI** | ***p* value** | **OR** | **95% CI** | ***p* value** | **OR** | **95% CI** | ***p* value** |
| Physical activity (minutes/day) | 1.00 | 0.99-1.00 | 0.41 |  |  |  | 1.00 | 0.99-1.00 | 0.50 | 1.00 | 0.99-1.00 | 0.44 |
| Frailty  Non-frail  Frail |  |  |  | Ref 1.21 | 0.63-2.32 | 0.57 | Ref 1.26 | 0.32-4.94 | 0.74 | Ref 0.61 | 0.25-1.47 | 0.27 |
| Physical activity * frailty |  |  |  |  |  |  | 0.99 | 0.96-1.02 | 0.34 |  |  |  |
| Age |  |  |  |  |  |  |  |  |  | 1.01 | 0.96-1.07 | 0.72 |
| Sex  Men  Women |  |  |  |  |  |  |  |  |  | Ref 1.89 | 0.98-3.63 | 0.056 |

**OR Odds ratio, CI Confidence Interval, Ref Reference group.**
